# Editorial: Equity in cancer care: mechanisms and interventions for enhanced access

**DOI:** 10.3389/fonc.2026.1934435

**Published:** 2026-07-31

**Authors:** Anna Santos Salas, Anna Pujadas Botey, Michelle Ann Eala

**Affiliations:** 1 Faculty of Nursing, University of Alberta, Edmonton, AB, Canada; 2 Research, Partnerships and Innovation, Cancer Care Alberta, Calgary, AB, Canada; 3 School of Public Health, University of Alberta, Edmonton, AB, Canada; 4 Department of Radiation Oncology, University of California, Los Angeles, Los Angeles, CA, United States

**Keywords:** cancer, cancer care access, health care services accessibility, health equity, interventions and programs, underserved communities

Cancer is a major global challenge, with incidence projected to rise in many regions (1). The impact of a cancer diagnosis goes beyond the individual and affects families and communities, who bear the physical, mental, and economic burden of cancer, that worsens with the rising costs of care and possible loss of income and employment. Importantly, a cancer diagnosis can trigger, expose, and amplify existing social and structural inequities that can lead to differences in people’s health and health outcomes. These differences are called inequities because they are avoidable, systematic, and unfair (2). Inequities in cancer care are reflected in underscreening and increased rates of late stage cancer diagnoses and cancer mortality, among others, and disproportionately affect underserved communities (3). These communities experience a greater share of cancer inequities due to persistent social and health disadvantages shaping access to care, quality of care, and cancer outcomes (4).

The overall goal of this research topic was to develop an informed understanding of the structural causes of cancer inequities and actionable strategies to alleviate them. By focusing on underserved communities, we sought to uncover and address the structural determinants and practical barriers these groups face in accessing equitable cancer care. Contributors to this research topic advance knowledge of how inequities in cancer care come to be in diverse world regions, report interventions or programs addressing these, and outline important recommendations.

Authors present evidence of factors contributing to inequities as well as innovative models or strategies to tackle these. In their review, Rodrigues dos Anjos et al., examine survivorship care in low-and-middle income countries highlighting critical challenges cancer survivors in these countries face as well as the need for innovative community-based models such as Brazil’s Mais Médicos program. In a systematic review comparing countries of different economies, Yilihamu and Aierxiding compare cancer education systems across countries with different economic contexts and identify important gaps in cancer education policy, resources, and public awareness among affluent and less affluent countries. Koukoutzeli et al., explore the critical role of time to reimbursement in access to novel anticancer drugs in the European Union, and describe the BEACON project, an initiative that promotes intersectoral and cross country collaboration throughout the cancer continuum to improve access.

Through diverse methodological approaches, contributors examined factors shaping cancer care and how health systems can advance cancer care equity. Courtney at al., examine the role of neighborhood characteristics on disparities in radiation oncology service utilization in the United States, showing both socioeconomic differences in the use of radiotherapy technique as well as potential contrasts with existing evidence in access to radiation oncology services. Horrill et al., describe dimensions to promote equity-oriented cancer care such as adopting core values, commitments to resources, leaders, and partners, and accountability and concrete strategies for those delivering cancer care.

Interventions and strategies to address gaps were also reported by contributors. Savas et al., show how a community health worker education program and navigation can improve access to breast or cervical cancer screening for underserved Latinas. Santos Salas et al., propose the development of a patient-oriented health-equity pathway to improve equity and wellness in cancer care with racialized communities in Canada, highlighting the significance of community engagement and patient-oriented research. Al Madhi et al., discuss the need for intersectoral collaboration, development of stronger data systems, and incorporating patient perspectives to improve access to cancer care and address inequities in the Middle East and African region.

In sum, research topic contributors clearly articulate factors and mechanisms underlying inequities in cancer care across diverse world settings, outline principles and practices to advance equity in cancer care, and describe effective interventions, strategies, and programs to improve equity. In today’s cancer care context, equity is widely recognized as a pressing goal so that so that all people, particularly those experiencing social and health disadvantage, have fair opportunities to benefit from advances. The works reported in this research topic show that it takes a concerted and collective effort to achieve equity. These works show that it is possible. We conclude with the words of critical educator Paulo Freire, ‘*change is difficult but possible*’ (5). Achieving equity is difficult but possible.
